# The olfactory system as the gateway to the neural correlates of consciousness

**DOI:** 10.3389/fpsyg.2013.01011

**Published:** 2014-01-10

**Authors:** Christina Merrick, Christine A. Godwin, Mark W. Geisler, Ezequiel Morsella

**Affiliations:** ^1^Department of Psychology, San Francisco State UniversitySan Francisco, CA, USA; ^2^School of Psychology, Georgia Institute of TechnologyAtlanta, GA, USA; ^3^Department of Neurology, University of California San FranciscoSan Francisco, CA, USA

**Keywords:** olfaction, consciousness, neural correlates of consciousness, conscious perception, olfactory consciousness

## Abstract

How consciousness is generated by the nervous system remains one of the greatest mysteries in science. Investigators from diverse fields have begun to unravel this puzzle by contrasting conscious and unconscious processes. In this way, it has been revealed that the two kinds of processes differ in terms of the underlying neural events and associated cognitive mechanisms. We propose that, for several reasons, the olfactory system provides a unique portal through which to examine this contrast. For this purpose, the olfactory system is beneficial in terms of its (a) neuroanatomical aspects, (b) phenomenological and cognitive/mechanistic properties, and (c) neurodynamic (e.g., brain oscillations) properties. In this review, we discuss how each of these properties and aspects of the olfactory system can illuminate the contrast between conscious and unconscious processing in the brain. We conclude by delineating the most fruitful avenues of research and by entertaining hypotheses that, in order for an olfactory content to be conscious, that content must participate in a network that is large-scale, both in terms of the neural systems involved and the scope of information integration.

## INTRODUCTION

How consciousness is generated by the nervous system remains one of the greatest mysteries in science (Crick and Koch, 2003; Roach, 2005): “No one has produced any plausible explanation as to how the experience of [anything]… could arise from the actions of the brain” (Crick and Koch, 2003, p. 119). Researchers from diverse fields have begun to unravel this puzzle by contrasting conscious and unconscious processes (Shallice, 1972; Logothetis and Schall, 1989; Crick and Koch, 1995; Kinsbourne, 1996; Wegner and Bargh, 1998; Grossberg, 1999; Di Lollo et al., 2000; Dehaene and Naccache, 2001; Baars, 2002, 2005; Gray, 2004; Libet, 2004; Laureys, 2005; Morsella, 2005; Merker, 2007; Doesburg et al., 2009; Damasio, 2010; Boly et al., 2011; Panagiotaropoulos et al., 2012). Through this contrastive approach, it has been revealed that the two kinds of processes differ in terms of the underlying neural events and associated cognitive mechanisms (see conclusions of this contrast in Godwin et al., 2013). (For discussion of the limitations of contrastive approaches, see Aru et al., 2012.) It has been proposed that, for several reasons, the olfactory system provides a unique portal through which to examine this contrast (Morsella et al., 2010; Keller, 2011). For this purpose, the olfactory system is beneficial in terms of its (a) neuroanatomical aspects, (b) phenomenological and cognitive/mechanistic properties, and (c) neurodynamic (e.g., brain oscillations) properties. In the three sections below, we discuss how each of these properties and aspects of the olfactory system can illuminate the contrast between conscious and unconscious processing in the brain.

## NEUROANATOMY

When reverse engineering a complex phenomenon, it is best to focus on the simplest manifestation of that phenomenon. For example, when investigating the neural correlates of consciousness, it is more fruitful to focus on primitive states such as pain, the perception of a tone, or the smell of a rose than to focus on more elaborate and, in terms of cognitive processing, more multifaceted states, such as nostalgia and, say, an appreciation of the narrative structure of a novel. From this reductionistic standpoint, we propose that the best system for investigating the neuroanatomical correlates of consciousness is that of olfaction (Freeman, 2007a; Freeman and Quian Quiroga, 2013). To appreciate this proposal, it is necessary to first apprehend the neuroanatomy of olfaction. Hence, we now present a brief, selective review of the neuroanatomy of the olfactory system, with an emphasis on the regions most pertinent to the study of consciousness. (For a more thorough review of the olfactory system, see Neville and Haberly, 2004; Shepherd et al., 2004.)

Olfaction, perhaps the phylogenetically oldest sensory modality (Hosek and Freeman, 2001), is unique among sensory modalities in its anatomical organization (Price, 1990; Freeman, 2007a). Most notably, unlike other sensory modalities (e.g., vision, audition, or touch), bottom-up afference from the olfactory receptors bypasses the thalamic “first-order” relay neurons (Sherman and Guillery, 2006) and directly influences a region of the ipsilateral cortex (Shepherd and Greer, 1998; Tham et al., 2009), called the olfactory (piriform) cortex (Haberly, 1998; Mori et al., 1999; Neville and Haberly, 2004; Gottfried and Zald, 2005). Specifically, after sensory transduction in the olfactory epithelium of the nose, olfactory afference undergoes sophisticated processing in the olfactory bulb, a structure that can generate complex patterns of activation across neural populations, which are used for the encoding of odorants (Freeman, 1987; Xu et al., 2000). While historically the olfactory bulb was compared to the retina (Ramón y Cajal, 1909–1911), it has been proposed more recently that the primary function carried out by the bulb is similar to the primary function carried out by the first-order relay thalamus (e.g., the lateral geniculate nucleus) in other sensory modalities (e.g., vision): “both structures act as a bottleneck that is a target for various modulatory inputs, and this arrangement enables efficient control of information flow before cortical processing occurs” (Kay and Sherman, 2007, p. 47).

After processing in the bulb, olfactory afference is processed in the piriform (meaning, “pear-shaped”) cortex. The piriform cortex is considered to be part of the “primary olfactory cortex,” which also includes the olfactory tubercle, the periamygdaloid cortex, the lateral entorhinal cortex, the cortical portion of the amygdaloid nuclei, the ventral tenia teat, and the nucleus of the lateral olfactory tract (Carmichael et al., 1994; Tham et al., 2009). Piriform cortex is a phylogenetically old type of cortex, hence the namesake of this kind of cortex, *paleocortex*. Paleocortex contains only three cortical layers, which stands in contrast to neocortex, which contains six layers. (It is worth noting that the analogous cortical regions for the modalities of vision and audition consist, not of paleocortex, but of neocortex.) Interestingly, though paleocortex is less complex than neocortex, it still shares remarkable similarities with the neocortex, in terms of physiology, neurochemistry, and local circuitry (Haberly, 1998). Thus, by studying this possibly more simple form of cortex, one can learn a great deal about neocortex.

Despite the relative simplicity of the piriform cortex, it has been suggested that the anatomical connectivity of the posterior piriform may allow it to perform complex operations such as learning, memory retrieval, and other associative functions (Haberly, 1998). Indeed, a study of odor learning in humans revealed that learning-induced neural plasticity is observed in the posterior piriform cortex in a fashion similar to that found in a higher cortical region involved in olfaction, the orbitofrontal cortex (OFC; Li et al., 2006). The piriform cortex may also have a role in the seeming stability of odor perception through stimulus generalization (Barnes et al., 2008; Sela and Sobel, 2010). Ensembles of neurons in the piriform cortex respond similarly to a mix of odors and to the same mix of odors when one odor is removed, but they will respond differently if one of the odors is replaced by a novel odor (Barnes et al., 2008).

Two main output pathways carry odor information from the piriform cortex to other brain regions. The first output pathway targets subcortical limbic regions (e.g., the hypothalamus) that are involved in motivational, emotional, and homeostatic responses to odors. The second output stream from the piriform cortex targets neocortex (Tham et al., 2009). This output stream to the neocortex can be further broken down into two distinct pathways (Tanabe et al., 1975). The primary (direct) pathway projects from pyramidal cells in the piriform cortex directly to the OFC and is considered the chief pathway for odor information to be transmitted to neocortical areas (Yarita et al., 1980; Carmichael et al., 1994; Haberly, 1998). The secondary (indirect) pathway originates from a relatively small number of cells in the piriform cortex and projects to the OFC through the mediodorsal thalamic nucleus (MDNT). This pathway consists of only sparse fiber density (Price and Slotnick, 1983; Haberly, 1998; Ongür and Price, 2000; see also Poo and Isaacson, 2009).

As noted, the indirect pathway, involving the MDNT, has sparse fiber density compared to the direct monosynaptic pathway. Despite its sparse fiber density, there is evidence that this pathway may be involved in significant olfactory processing. For example, patients with damage to the MDNT show deficits in odor identification, discrimination, and hedonics (Potter and Butters, 1980; Sela et al., 2009). Furthermore, bilateral thalamic infarctions yield sudden, transient abnormalities in consciously experienced odor perception (Asai et al., 2008). There has also been an argument for the involvement of the MDNT in olfactory attention (Plailly et al., 2008). Based on these findings, one can tentatively conclude that the MDNT is neither necessary nor sufficient for conscious olfactory experience, but that it may play a role in olfactory identification, discrimination, and hedonics, as well as in the orienting of olfactory attention.

The OFC is the principal neocortical region for olfactory processing. It performs associative roles in olfactory information processing (Gottfried and Zald, 2005) and carries out multisensory integration (Rolls and Baylis, 1994). For example, it is in the OFC that inputs from gustation, olfaction, somatosensation, audition, and vision combine to create the multimodal perception of flavor (Rolls and Baylis, 1994; Shepherd, 2006). The OFC seems to play a particularly important role in primate cognition (Tanabe et al., 1975) and occupies a role in “central processing.” It has been demonstrated that the magnitude of OFC activation (but not that of piriform cortex) predicts the degree of subsequent improvement in an olfactory judgment task (Li et al., 2006).

In summary, unlike most other sensory modalities, afferents from the olfactory sensory system (a) bypass the first-order, relay thalamus, (b) directly target the cortex ipsilaterally (Shepherd and Greer, 1998; Tham et al., 2009), which minimizes spread of circuitry, (c) involve a primary processing area that consists of paleocortex (which contains only half of the number of layers of neocortex), and (d) involve primarily only one brain region (the frontal cortex; Shepherd, 2007). The last observation stands in contrast to vision and audition, which often involve large-scale interactions between frontal cortex and parietal cortices, as in the case of the well-documented interactions between frontal-parietal cortex or frontal-occipital cortex. This summary reveals the relative simplicity of the anatomy of the olfactory system compared to that of other systems. In addition, it has been claimed that, because of its positioning within the cranium, the olfactory system features a privileged and accessible region (Shepherd and Greer, 1998). As Shepherd (2007) concludes, “In olfactory perception there is no ‘back’ of the brain; the primary neocortical receptive area is in the OFC, which is at the core of the prefrontal area. Thus, in olfaction, all of the sequences of processing that are necessary to get from the back to the front of the brain are compressed within the front of the brain itself. This reflects the evolutionary position of smell, with its privileged input to the highest centers of the frontal lobe throughout the evolution of the vertebrate brain. From this perspective, the basic architecture of the neural basis of consciousness in mammals, including primates, should be sought in the olfactory system, with adaptations for the other sensory pathways reflecting their relative importance in the different species” (p. 93).

### IMPLICATIONS FOR CONSCIOUSNESS

We now discuss the conclusions that can be drawn regarding the neuroanatomy of olfactory consciousness. First, we discuss the role of the most peripheral anatomical structures: the olfactory epithelium and olfactory bulb. Regarding the latter, previous findings suggest that the olfactory bulb is unnecessary for endogenic olfactory consciousness (Mizobuchi et al., 1999; Henkin et al., 2000). (Again, the bulb has been described as being functionally equivalent to the first-order relay of the thalamus; Kay and Sherman, 2007; see also Murakami et al., 2005.) This observation is consistent with findings from research on the neural correlates of various kinds of conscious olfactory experiences, including olfactory perceptions, olfactory imagery, and olfactory hallucinations (Markert et al., 1993; Mizobuchi et al., 1999; Leopold, 2002). This research, which includes neuroimaging studies (Henkin et al., 2000), experiments involving direct stimulation of the brain (Penfield and Jasper, 1954), and lesion studies (Mizobuchi et al., 1999), suggests that endogenic, olfactory consciousness does not require the olfactory bulb. Perhaps most critically, it seems that patients with bilateral olfactory bulbectomies can still experience explicit, olfactory memories, suggesting that, under certain circumstances, these peripheral structures are not necessary for the instantiation of these kinds of conscious representations. However, the current literature lacks systematic, conclusive studies regarding this important clinical observation.

It is worth noting that Kallmann Syndrome, a genetic disorder in which the olfactory bulb and its tracts develop abnormally, is often characterized by complete anosmia or hyposmia (Madan et al., 2004; Fechner et al., 2008). Similarly, bifrontal craniotomies, a surgical procedure that removes the olfactory bulbs or olfactory nerves, have been performed on patients with severe phantosmias (e.g., olfactory hallucinations) and have yielded bilateral permanent anosmia (Markert et al., 1993). Excision of the olfactory epithelium has also been used as a treatment for severe phantosmias. In some cases, the procedure is not only effective in eliminating the phantosmias, but the patient has his/her olfactory ability restored after some time (Leopold, 2002). Based on these findings, one can conclude that, though there is some evidence that olfactory consciousness of some kind can persist despite the absence of the olfactory epithelium and olfactory bulb, more data are required before drawing strong conclusions regarding the necessary role of these peripheral structures in olfactory consciousness.

Second, we discuss the role of the thalamus. Although in olfaction the thalamus is not immediately influenced by the bottom-up afference (as is the case in other modalities), the MDNT does receive inputs from cortical regions that are involved in olfactory processing (Haberly, 1998). Hence, one should refrain from concluding that, in olfactory consciousness, thalamic processing is unnecessary. Nevertheless, because olfactory afferents bypass the relay thalamus, one can draw a more conservative conclusion: Consciousness of some sort does not require the first-order thalamic nuclei, at least for olfactory experiences and under several assumptions (Morsella et al., 2010).

It is likely that the MDNT plays a significant role in high-level olfactory processes, those above the processing of the early afferent signal. For example, as mentioned above, evidence suggests that this structure is important in olfactory discrimination (Eichenbaum et al., 1980; Slotnick and Risser, 1990; Tham et al., 2011), olfactory identification, and olfactory hedonics (Sela et al., 2009). The MDNT is also significant in more general cognitive processes, including attentional mechanisms (Tham et al., 2009, 2011), learning (Mitchell et al., 2007), and memory (Markowitsch, 1982). It is important to note that, pertinent to the topic at hand, no study we are aware of has documented a lack of basic conscious olfactory experience resulting from lesions of the MDNT (but see theorizing in Plailly et al., 2008). It seems that olfactory discrimination of some sort can survive following lesions of the MDNT (Slotnick and Kaneko, 1981; Slotnick and Risser, 1990).

In addition, it is important to consider that, regarding second-order thalamic relays such as the MDNT, these nuclei are similar in nature to first-order relays in terms of their circuitry (Sherman and Guillery, 2006). Thus, the circuitry of the MDNT is quite simplistic compared to, say, that of a cortical column. In addition, as mentioned above, the thalamus in olfactory processing involves only sparse fiber density. One might propose that such simplistic circuitry would be insufficient to instantiate a phenomenon as sophisticated as consciousness, but such a conclusion would be premature. To date, there is no strong theorizing regarding the kind of circuitry that the instantiation of consciousness would entail. Moreover, very little is known about all aspects of thalamic processing (see Morecraft et al., 1992). Hence, at this stage of understanding, one cannot rule out that thalamic processes are capable of constituting consciousness (see strong evidence for involvement of the thalamus in consciousness in Merker, 2007; Ward, 2011).

Regarding the paleocortex, it has been proposed that cortical involvement is required for consciousness of any kind (see various accounts in Godwin et al., 2013). Thus, lesions of the cortical regions involved in olfactory processing should result in the inability to have conscious olfactory experiences. According to Barr and Kiernan (1993), olfactory consciousness depends primarily on the piriform cortex. It is interesting to note that, if conscious olfactory experience can arise at the level of the piriform cortex, then this would be the only case in which a sensory system achieves conscious perception with little or no involvement of neocortical or thalamocortical circuits. However, according to Sela and Sobel (2010), and based on our own review of the literature, there are no documented cases of anosmia that have arisen due to focal lesions of the piriform cortex. Accordingly, in the animal literature, Staubli et al. (1987) showed that rats with ablations to the piriform cortex were still able to discriminate between simple odor cues (although not complex odor cues) in a comparable manner to control rats, suggesting that the piriform cortex may aid in more complex odor discrimination tasks but is unnecessary for the discrimination between simple odors. (Of course, one must be conservative when drawing conclusions about the conscious experience of these animals.)

Complementing these observations, the piriform cortex exhibits increases in odorant-induced activity at the onset of a new odor (Sobel et al., 2000; Poellinger et al., 2001). Although the time-course of this activation (from fMRI studies) varies from study to study [from 10 to 15 s (Poellinger et al., 2001) and 30 to 40 s (Sobel et al., 2000)], both the studies by Poellinger et al. (2001) and Sobel et al. (2000) indicate that accurate odor detection persists after activation in the piriform decreases to a baseline (or below baseline) level. Conversely, activation in the OFC does not decrease over odorant exposure (60 s; Poellinger et al., 2001). This difference in activation levels may represent the functional importance of olfactory tracts that bypass the piriform cortex and project directly from the bulb to the OFC (Shipley and Adamek, 1984).

Regarding the role of neocortex, Keller (2011) concludes, “There are reasons to assume that the phenomenal neural correlate of olfactory consciousness is found in the neocortical OFC” (p. 6; see additional evidence in Mizobuchi et al., 1999). In line with this proposal, Cicerone and Tanenbaum (1997) observed complete anosmia in a patient with a lesion to the left orbital gyrus of the frontal lobe, and Li et al. (2010) reported a comprehensive case study of a patient who experienced complete anosmia as a result of a right OFC lesion. Despite the patient’s complete lack of conscious olfactory experience, neural activity and autonomic responses revealed a robust sense of *blind-smell* (unconscious olfactory processing that influences behavior; Sobel et al., 1999), a phenomenon we discuss below. This evidence suggests that, while many aspects of olfaction can occur unconsciously, the OFC is necessary for conscious olfactory experience. Independent of this research, and consistent with cortical accounts of consciousness, it has been proposed that the conscious aspects of odor discrimination depend primarily on the activities of the frontal and orbitofrontal cortices (Buck, 2000).

However, not all accounts implicate the neocortex in the generation of olfactory consciousness (e.g., Barr and Kiernan, 1993) and not all documented lesions of the OFC have resulted in anosmia. For instance, Zatorre and Jones-Gotman (1991) documented cases in which OFC lesions resulted in severe deficits, yet all patients demonstrated normal olfactory detection thresholds. Zatorre and Jones-Gotman (1991) conclude that the OFC is important in odor discrimination but not in conscious odor detection. Moreover, in the animal literature, rats with lesions of the OFC still perform normally on odor-identification tasks (Tait and Brown, 2007). Of course, only limited conclusions can be drawn because of the neuroanatomical differences in the OFC between the rat and humans (Uylings et al., 2003) and because of the difficulty of determining whether the animal is consciously experiencing a smell (e.g., the behavior of the animal could reflect a sort of blind smell).

In conclusion, although it is clear that the olfactory system is well suited system for the isolation of a neural correlate of consciousness, the current literature does not permit one to draw strong conclusions regarding the neuroanatomical regions that are critical for the generation of olfactory consciousness. Investigations on the neural correlates of phantosmias may further illuminate the circuits required for olfactory consciousness. But this is challenging research: It has proven difficult to identify the minimal region(s) whose stimulation is sufficient to induce olfactory hallucinations (Mizobuchi et al., 1999). It appears that, once more data are available, conclusions with greater certainty may soon be drawn, especially concerning the roles of the peripheral structures (the olfactory epithelium and bulb) and the MDNT in the generation of olfactory consciousness.

## PHENOMENOLOGICAL AND COGNITIVE/MECHANISTIC PROPERTIES

There are phenomenological and cognitive/mechanistic properties that render the olfactory system a fruitful network in which to investigate the contrast between conscious and unconscious processing. Regarding the phenomenological properties, unlike what occurs with other sensory modalities, olfaction regularly yields no subjective experience of any kind when the system is under-stimulated, as when odorants are in very low concentrations or during sensory habituation to odorants. This “experiential nothingness” (Morsella et al., 2010) is more akin to the phenomenology of the blind spot than to what one experiences when visual stimulation is absent (darkness). It is important to note that, in the latter case, there still exists a conscious, visual experience (e.g., that of a black field). The experiential nothingness produced by an olfactory system yields no conscious contents of any kind to such an extent that, absent memory, one would not be able to know that one possessed an olfactory system. (For a comparison of olfactory consciousness to the phenomenon of *change blindness* in vision, see Sela and Sobel, 2010.)

As noted, this form of experiential nothingness can result from habituation or from an inadequate level of olfactory stimulation. In the latter case, subjects may be consciously unaware of the presence of an odorant (e.g., lavender) but still be influenced by the odorant unconsciously, as in the phenomenon of blind-smell (Sobel et al., 1999), the olfactory analog of *blindsight* (Weiskrantz, 1992), in which patients report to be blind but still exhibit visually guided behaviors. For example, in blindsight, a patient may self-report to be unable to see anything but may nonetheless walk around an obstacle placed in her path. In blind-smell, people can learn to associate certain odorants (e.g., lavender) with certain environments (e.g., a particular room), even though the concentration of odorants presented during learning was consciously imperceptible (Degel et al., 2001). That the subliminal odorant is influencing behavior is detectable in behavior and decision-making. The findings from research on blind-smell complement similar findings from investigations on subliminal visual perception (Pessiglione et al., 2007; see review in Hallett, 2007). Thus, the olfactory system features properties that render it ideal for experiments designed to contrast the neural correlates of sensory processes that are conscious (e.g., the smell of fresh bread) with those that lie in an experiential nothingness, as in the blind-smell of subliminal odorants.

Regarding habituation, though this phenomenon occurs in all sensory modalities, it may occur in a special manner for olfaction because of the absence of the possibility of voluntary re-access to an exposed odorant (Stevenson, 2009). For example, in haptic sensation, one may habituate to the feeling of wearing a wrist watch. Similar habituation can occur in olfaction: Upon entering a room, one may detect a smell for some time, before one habituates, and then the smell vanishes from consciousness. Although both sensory stimuli fade from consciousness, the feeling of one’s watch can be experienced anew by voluntarily directing attention toward the watch. However, it seems that olfactory sensations cannot be re-accessed as easily through these attentional means (Köster, 2002). Regarding the experiential nothingness associated with olfaction, it is important to reiterate that research indicates that (a) accurate odor detection persists after activation in the piriform decreases to a baseline (or below baseline) level (Sobel et al., 2000; Poellinger et al., 2001), and (b) activation in the OFC does not decrease over odorant exposure (60 s; Poellinger et al., 2001).

**FIGURE 1:**
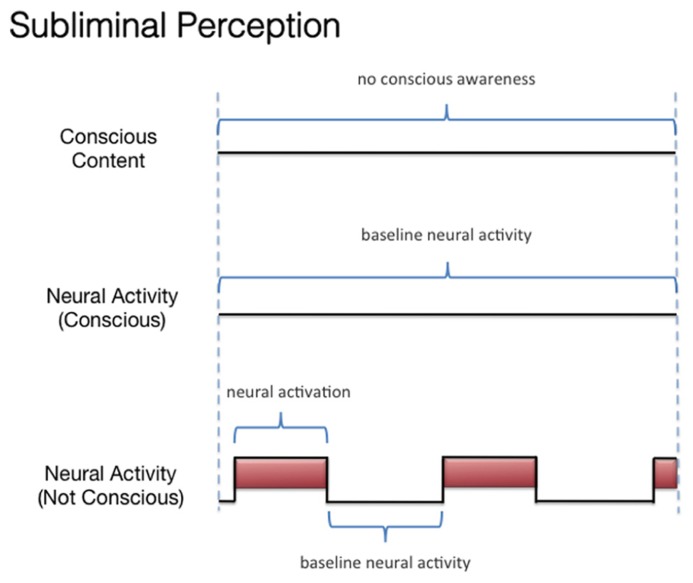
**Schematic representation of the relationship between the subjective events associated with subliminal perception of olfactory stimuli and hypothetical neural activity**.

**FIGURE 2:**
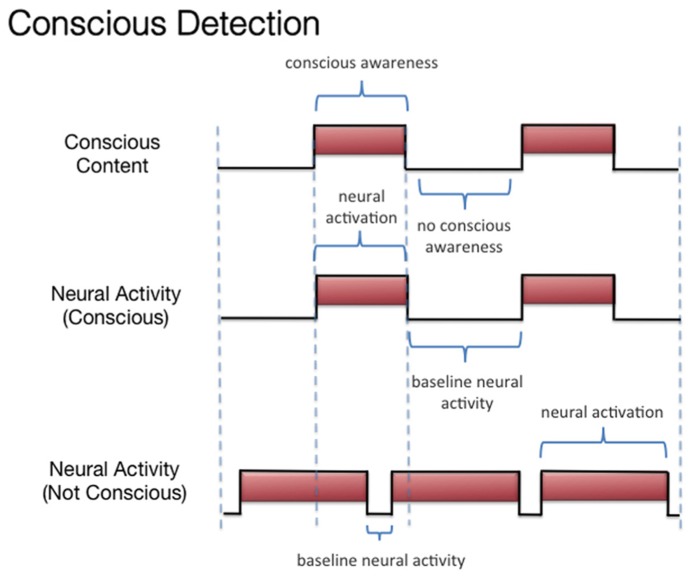
**Schematic representation of the relationship between the subjective events associated with conscious detection of olfactory stimuli and hypothetical neural activity**.

**FIGURE 3:**
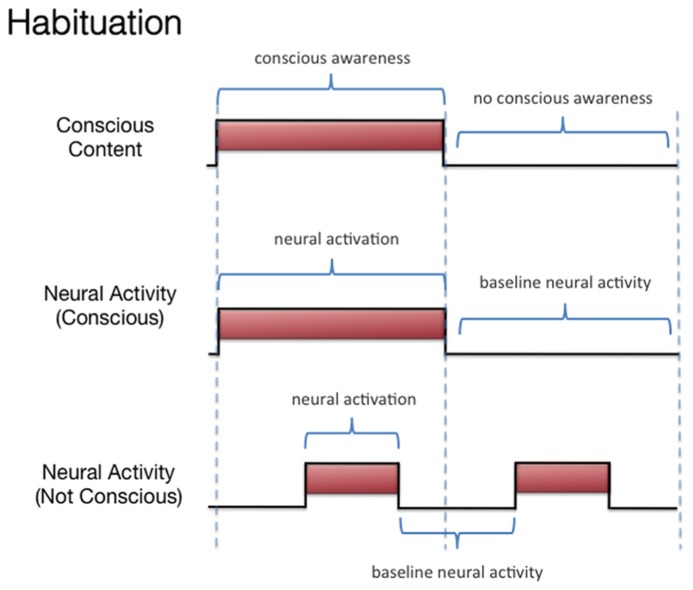
**Schematic representation of the relationship between hypothetical neural activity and the subjective events associated with habituation of olfactory stimuli**.

We discussed three states associated with olfactory consciousness: (A) subliminal perception (**Figure 1**), which includes no conscious contents, (B) conscious detection of an odorant (**Figure 2**), which includes conscious contents and is indexed by self-report on the part of the subject, and (C) habituation (**Figure 3**), which, like subliminal perception, includes no conscious contents. When isolating the neural correlate of olfactory consciousness (NCC-O), one should seek regions that are active during B but not during A and C. It is important to note that the NCC-O of an odorant, as indexed by self-report, should not vary as a function of the organism’s motivational or incentive state.

Consider the example in which a master chef must detect whether the soups served in her restaurant are being cooked properly. The chef must compare the smell dimension of the dish to some standard in memory (e.g., perfect carrot soup). This conscious perception occurs in an invariable manner regardless of the chef’s current emotional/incentive state. This is obvious when one observes in the chef a series of invariable judgments made when evaluating soups at different times. It would not be adaptive for the smell of token odorants (e.g., a soup) to be identified differently at different times, because of variables concerning the internal state of the organism. Of course, if the chef is hungry or sated, her entire conscious experience will be different when smelling the food item, but these motivational/incentive variables reflect other dimensions of conscious experience. Simply put, the smell of a banana, if experienced consciously by the organism, must be experienced in the same way when the organism is, say, hungry or sated. It is adaptive for there to be such an invariance and an independence between motivation and perception, as noted by Rolls et al. (1977) in their discussion of the limits of motivational influences over visual perception: “It would not be adaptive, for example, to become blind to the sight of food after we have eaten it to satiety” (p. 144). This is because food items are not used just for eating; they can also be used as, say, projectiles to throw at an entertainer.

**FIGURE 4:**
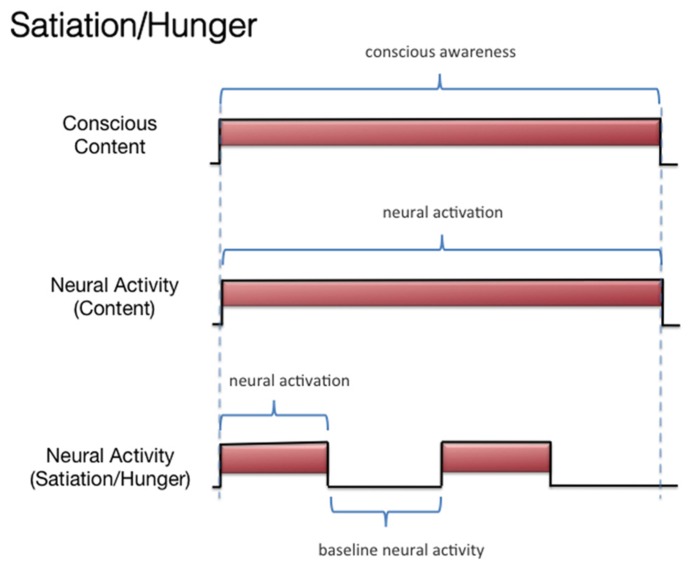
**Schematic representation of the relationship between subjective olfactory experience and hypothetical neural activity, under conditions of hunger and satiation**.

Hence, the perception of the items should be invariant and not vary by emotional/incentive state. Our chef example reminds one of the multidimensional nature of conscious experiences and is reminiscent of the classic research regarding the multiple conscious dimensions of subjective pain (e.g., the sensorial and affective dimensions; Melzack and Casey, 1968). To give another example, no one who “grew to like olives,” who at first did not like olives, ever thought that the first olive they ever tasted failed to represent subsequent olives, at least in terms of the flavor. When *growing to like* olives, something does change in one’s conscious experience, but it is not the conscious perception of the olive flavor. This has implications for the study of the NCC-O: The NCC-O for a given odorant should vary as a function of whether there is (A) subliminal perception, (B) conscious detection of an odorant, or (C) habituation. However, it should not vary as a function of the organism being sated or hungry (**Figure 4**). It should be clarified, however, that it remains an empirical question whether the NCC-O of an odorant remains unchanging regardless of, say, the organism’s current incentive/motivation state or the positive/negative contingencies associated with that odorant. There is evidence suggesting that the neural pattern underlying the representation of an odorant is changed slightly if that odorant is reinforced or unreinforced through conditioning (Freeman, 2007b; see also Keller, 2011).

Concerning habituation, its effects can be seen at the receptor level as well as at the level of the olfactory bulb (Wilson, 2009). Data concerning the functioning of the piriform cortex and OFC during habituation are less straightforward (Sobel et al., 2000; Poellinger et al., 2001; Wilson, 2009).

The olfactory system is a fruitful network in which to isolate the NCC-O also because of its cognitive/mechanistic properties. First, unlike in the visual modality, there are few sophisticated cognitive control functions that are usually coupled with olfactory processing. For example, there is no phenomenon in olfaction that is analogous to mental rotation, a form of high-level symbol manipulation. Thus, in olfaction, one is less likely to conflate the NCC-O with the neural correlates of high-level executive functions (see Aru et al., 2012), which is a recurring problem in the search for the visual NCC (see discussion in Panagiotaropoulos et al., 2013). In addition, because of the relative lack of control functions in olfaction, the subjective experience of the self-reporting subject is unlikely to be contaminated by introspections regarding, not olfactory experience, but cognitive effort or other aspects of control. In a similar vein, in vision and audition, mental images can be used to preserve information in working memory through active executive processes such as rehearsal (Baddeley, 2007), but olfactory images are difficult to couple with such executive operations (Stevenson, 2009). In fact, it has been demonstrated that participants report that olfactory images are more difficult to produce and less vivid in comparison to other forms of mental imagery (Betts, 1909; Brower, 1947; Lawless, 1997; Stevenson, 2009).

Second, olfactory experiences are less likely to occur in a self-generated, stochastic manner. Unlike in the case of vision and audition, in which visually rich daydreaming or “ear worms” (i.e., a song involuntarily repeating in one’s head) can occur spontaneously during an experiment and contaminate visual and auditory dependent measures, respectively, there are little, if any, such self-generated olfactory experiences that could contaminate psychophysical measures. Last, the olfactory system is more segregated from the semantic system than is the most studied sensory system – vision. Many have argued that, in the case of vision, there are deep, inextricable relationships among perception, conceptualization, and semantic processing (Barsalou, 1999; Kosslyn et al., 2006). Such is not the case for olfaction. Thus, when isolating the NCC-O, one is less likely to include in it higher-level processes (e.g., semantic processes) that are associated with more than just simple olfactory (conscious) detection.

## NEURODYNAMICS

For present purposes, it is fortunate that the olfactory system was one of the first systems in which the nature of oscillatory activity in the brain was investigated (e.g., Adrian, 1942). Before discussing research on the nature of this oscillatory activity, we will discuss the more general relationship between brain rhythms and consciousness.

It has been proposed that, to instantiate consciousness of any kind, the mode of interaction among regions is as important as the nature and loci of the regions activated (Ward, 2003; Buzsáki, 2006; Godwin et al., 2013). For example, the presence or lack of *interregional synchrony* leads to different behavioral, cognitive, and even consciously experienced outcomes (Ward, 2003; Hummel and Gerloff, 2005; Lewis et al., 2012). [See review of neuronal communication through “coherence” in Fries (2005)]. Regarding the neurodynamics underlying consciousness, the general view is that consciousness depends on “precise synchronization of oscillatory neuronal responses in the high frequency range (beta, gamma)” (Singer, 2011, p. 43). Singer (2011) adds, “brain states compatible with conscious processing should be characterized by a high degree of synchrony” (p. 43). Similar conclusions about the role of high frequencies in consciousness are found in the literature (e.g., Crick and Koch, 1990; Engel and Singer, 2001; Meador et al., 2002; Jung-Beeman et al., 2004; Doesburg et al., 2005; Aru and Bachmann, 2009; Doesburg et al., 2009; Uhlhaas et al., 2009; Hameroff, 2010; Wessel et al., 2012). Most recently, with the use of more sensitive technologies, the hypothesis was supported by Panagiotaropoulos et al. (2012), who examined activities of the lateral prefrontal cortex of the macaque. As revealed below, the olfactory system has the potential to provide additional evidence for these conclusions, such as those concerning the roles of gamma and beta frequencies in cognition. In addition, the relative simplicity of the neuroanatomical architecture of the system renders it a fruitful environment in which to investigate the neurodynamics of consciousness. (For a general review of the role of oscillations in cognition, see Sauseng and Klimesch, 2008; Wang, 2010; Siegel et al., 2012.)

Generally, the empirical evidence suggests that olfactory information may be encoded through oscillating neural assemblies (Adrian, 1942, 1950a,b; Freeman, 1975; Eeckman and Freeman, 1990; Gray, 1994; Laurent and Davidowitz, 1994; Kim et al., 2006). Different odorants elicit different, complex spatial patterns across spatially distributed neural ensembles of the olfactory bulb (Freeman, 1987; Laurent and Davidowitz, 1994; Xu et al., 2000). The elements comprising these dynamic patterns of activation are not static, but can evolve dynamically over time (Freeman, 1987; Laurent, 1996).

Classic research on the olfactory bulb, for example, illuminates the occurrence of organized, high frequency activity (gamma in the rat, ranging from 40 to 100 Hz; Adrian, 1942, 1950a,b; Kay and Beshel, 2010) during the perception of odorants. These high-frequency gamma “bursts” appear to be coordinated with respiration, which is associated with a slower oscillatory cycle (*theta* in the rat: 2–12 Hz; Eeckman and Freeman, 1990; Rojas-Líbano and Kay, 2008; Kay et al., 2009). [Concerning theta, Kay et al. (2009) state, “In the olfactory system, theta oscillations track the respiratory cycle and range in waking rodents from 2 to 12 Hz, with frequencies above 4 Hz defined usually as sniffing” (p. 9). See Schroeder and Lakatos (2009) for a treatment of the role of the respiratory cycle in oscillations.] Specifically, each phasic gamma burst begins shortly after inspiration, terminates during expiration, and can be modulated (via increases in frequency and duration) by the presence of an odorant (Eeckman and Freeman, 1990). Adrian (1942) associated the gamma burst with increased inter-cellular activity (including excitation and inhibition between neurons) within the olfactory bulb, a view that has been corroborated by subsequent research (Rall and Shepherd, 1968; Mori and Takagi, 1978; Gray, 1994; Lagier et al., 2004; Kay et al., 2009). [See Buzsáki and Wang (2012) for discussion of the origins of gamma oscillations.] If the lateral olfactory tract–axons of a subset of cells from the bulb that project to the piriform cortex (Haberly, 1998)–is severed or otherwise disrupted, gamma oscillations in the bulb persist (Gray and Skinner, 1988), but gamma no longer occurs in piriform cortex (Freeman, 1979; Haberly, 1998). This suggests that the mechanism involved in producing gamma oscillations resides within the olfactory bulb. (For a treatment of the interactions between the olfactory bulb and cortex, see Boyd et al., 2012; Oswald and Urban, 2012. For research on the role of gamma as a “temporal filter,” see Litaudon et al., 2008.)

Further support for the aforementioned hypothesis that the olfactory bulb is the functional equivalent of the thalamus is provided by the study of oscillations in the olfactory system. Experiments have revealed that correlations between slow-wave (theta) activity in the olfactory bulb and piriform cortex resemble those found between the thalamus and neocortex (Fontanini et al., 2003; Fontanini and Bower, 2006). Importantly, local field potentials and intracellular membrane potentials in the piriform cortex are strongly correlated with the slow-wave oscillatory pattern of the olfactory bulb (Fontanini and Bower, 2006). A similar inter-relationship occurs between the thalamus and neocortex (Contreras and Steriade, 1995).

The functional role that gamma oscillations may play in olfaction and in sensory perception is still under debate, as is the nature of processing in the olfactory bulb (Gervais et al., 2007). Research suggests that the higher the *task demand* (e.g., fine discrimination of odors versus simple discrimination of odors), the higher the gamma amplitude will be in early perceptual processing (Beshel et al., 2007). For example, in the olfactory bulb of the rat, when fine odorant discriminations are required in a two-alternative choice task, there are high gamma amplitudes, independent of changes in the frequency bands of theta and beta (Beshel et al., 2007). Accordingly, disturbing gamma oscillations in invertebrates impairs the discrimination of similar odors (a high task demand), but does not impair the discrimination of dissimilar odors (a low task demand; Stopfer et al., 1997).

Gamma oscillations have been studied in the mammalian olfactory system since the time of Adrian. More recently, beta oscillations (~15–30 Hz in the rat; Kay et al., 2009; Kay and Beshel, 2010) have attracted attention. These oscillations have been observed in response to volatile odorants and organic solvents, and are found in the olfactory bulb, piriform cortex, entorhinal cortex, and hippocampus (Zibrowski and Vanderwolf, 1997; Vanderwolf and Zibrowski, 2001). Unlike gamma oscillations, oscillations in the beta range require participation of (at least) the piriform cortex (Neville and Haberly, 2003). Surgical interruption of the lateral olfactory tract eliminates beta oscillations in the olfactory bulb (Neville and Haberly, 2003), whereas, as mentioned above, gamma oscillations can persist following such an interruption (Gray and Skinner, 1988).

It has been hypothesized that the reciprocal interactions between the bulb and piriform cortex engender local field potential oscillations in the beta range (Neville and Haberly, 2003). Beta oscillation episodes last longer than those of gamma oscillations, usually spanning 2–4 inhalation cycles in the rat. These oscillations are specific to a given odorant and reset when a new odorant is presented (Lowry and Kay, 2007). Beta oscillations in the olfactory bulb and anterior piriform cortex of the rat typically develop over the first three or four exposures to a particular odorant. In the piriform cortex of the rat, beta oscillations have also been shown to have a gradual enhancement or sensitization over repeated presentations of odorants, which, for certain odors, can last up to several days (e.g., Vanderwolf and Zibrowski, 2001). Beta coherence between the olfactory bulb and the hippocampus also accompanies odor learning in a go/no-go task (Martin et al., 2007). These oscillations have also been associated with certain types of odor learning (Martin et al., 2004; Kay et al., 2009) and odor discrimination. A study conducted by Kay and Beshel (2010) examined the phase of beta in the olfactory bulb and the anterior and posterior piriform cortices of the rat as the animal performed a two-alternative odor discrimination choice task. These investigators found that beta oscillations in the olfactory bulb drove or “entrained” both areas of the piriform, suggesting that beta oscillations may serve the purpose of transmitting olfactory information from the olfactory bulb to higher order, more cognitive areas. Accordingly, sensory research outside of olfaction has found evidence that beta may be involved in sensory gating (Hong et al., 2008) or in large-scale coupling for sensorimotor integration (Freeman, 2007b; Siegel et al., 2012). In addition, Kay et al. (2009) propose that, “beta oscillations are associated with motor models, favoring this oscillation as a good substrate for long-distance communication” (p. 7). Together, these studies suggest that beta oscillations may serve as a mechanism to link the olfactory system to various subcortical and cortical areas for cognitive processing (e.g., short-term perceptual learning and memory formation). Consistent with this interpretation, it has been proposed that, though gamma frequencies can be observed in processing at primary sensory areas, when the sensory information becomes part of a wider network which includes activations from other sensory modalities, then the frequencies are in the beta range (Freeman, 2007b). This occurs in olfaction (Freeman, 2007b). It remains unclear whether the higher frequency oscillations (e.g., those in the gamma range) play an essential role in the instantiation of conscious content (e.g., olfactory content *X*) or whether such a content can be instantiated independently by the more global (and slower) frequency ranges (e.g., beta). The neuroanatomical evidence reviewed above suggests that the central processes can instantiate the conscious representations of sensorial content without the peripheral structures. These facts remain puzzling.

Researchers have also examined the possible relationships among the different frequency bands (including *cross-frequency phase synchronization*; Sauseng and Klimesch, 2008) in the olfactory system. For example, Ravel et al. (2003) examined the relationship between gamma and beta oscillations by recording local field potentials in the olfactory bulb while rats performed an olfactory discrimination task. During this task, there was decreased power in the gamma band and increased power in the beta band (Ravel et al., 2003). The same pattern of activation was even more notable in well-trained rats, with gamma now being significantly decreased in both duration and amplitude, and beta power being amplified twofold during odor sampling (Ravel et al., 2003). As concluded by Kay and Beshel (2010), “Beta and gamma oscillations are not simply different frequencies but also show some opposing effects in the olfactory network” (p. 836). In addition, theta coherence (reflecting the strength of interaction between the olfactory bulb and piriform cortex) has been shown to increase parametrically to odorant volatility in awake rats but not in urethane-anesthetized rats (Lowry and Kay, 2007). Theta band coherence may facilitate beta oscillations, which, as mentioned above, may be a key mechanism for transmitting information across the olfactory system (Lowry and Kay, 2007; Kay and Beshel, 2010).

This brief survey into the neurodynamics of olfaction reveals that the relative simplicity of the neuroanatomical architecture of the olfactory system renders it a fruitful network in which to study brain oscillations (Freeman, 2007a; Schroeder and Lakatos, 2009). Examination of the long-studied oscillatory properties of the olfactory system corroborates what has been observed in other sensory modalities (cf., Fries, 2005; Sauseng and Klimesch, 2008; Singer, 2011; Siegel et al., 2012): (a) the synchronizations of high frequencies (e.g., gamma) in local (e.g., olfactory bulb) afferent processing (von Stein and Sarnthein, 2000; Bruns and Eckhorn, 2004; Kay and Beshel, 2010), especially when the process is challenging (e.g., fine discrimination versus simple discrimination; Kay and Beshel, 2010), and (b) the synchronization at a somewhat slower frequency range (e.g., beta or theta) for integration with a larger-scale cognitive network (Kay et al., 2009; Kay and Beshel, 2010), the next topic of discussion.

## LARGE-SCALE NETWORK PROPERTIES

While it has been proposed that each of the sensory modules (e.g., for the perception of color, motion, and depth) can generate some form of conscious contents on its own (a “microconsciousness”; Zeki and Bartels, 1999), others have argued that, to become conscious, a content must become part of a broader, supra-modal network. More specifically, it has been proposed that, for olfactory perceptual information (“olfactory content,” for short) to become a conscious content, it must interact with other, traditionally non-olfactory regions of the brain (Cooney and Gazzaniga, 2003). For example, olfactory contents may be transformed into conscious contents once they influence processes that are semantic-linguistic (Herz, 2003) or motor (Mainland and Sobel, 2006). These views are consistent with a consensus regarding the function of conscious processing more generally – that conscious processes integrate neural activities and information-processing structures that would otherwise be independent (Baars, 1988, 1998, 2005, 2013; Tononi and Edelman, 1988; Damasio, 1989; Freeman, 1991; Srinivasan et al., 1999; Zeki and Bartels, 1999; Edelman and Tononi, 2000; Dehaene and Naccache, 2001; Llinás and Ribary, 2001; Varela et al., 2001; Clark, 2002; Ortinski and Meador, 2004; Sergent and Dehaene, 2004; Morsella, 2005; Del Cul et al., 2007; Kriegel, 2007; Merker, 2007; Doesburg et al., 2009; Uhlhaas et al., 2009; Boly et al., 2011; Koch, 2012; Tallon-Baudry, 2012; Tononi, 2012). (See reviews of the integration consensus in Baars, 2002, 2013, and in Morsella, 2005.) Consistent with the integration consensus, Uhlhaas et al. (2009) specify that the earliest signature of conscious processing is, “the precise phase locking across a widely distributed cortical network” (p. 11). According to Freeman (2004), the conscious representations of information from different sources, such as from the different sensory modalities, must at some level be similar in form in order for the information from each of these modalities to be integrated with that of the other modalities, thereby forming a polysensory Gestalt of the world. In addition, the form must permit interaction between perceptual and motor systems (Freeman, 2004) if there is to be perception-to-action translations. It has been proposed that these perceptual Gestalts arise in consciousness in a discontinuous manner, with each conscious moment reflecting one snapshot of ongoing integration, resembling the still images of a motion picture, which, when presented one after the other, produce the illusion of continuous motion (Freeman, 2004, 2007b; Koch, 2004). (To learn about the level of representation that characterizes conscious contents, see Freeman, 2007a.)

Moreover, in both perception-based research and action-based research, conscious processing has been associated with more integration than unconscious processing, in terms of the information integration involved and in terms of the neural processes involved. For example, in action-based research, it has been documented that actions de-coupled from conscious processing [e.g., in blindsight, anarchic hand syndrome (Marchetti and Della Sala, 1998), automatisms, and other neurological disorders] reflect less integration than their conscious counterparts, as if the actions are not influenced by the kinds of information by which they should be influenced. Hence, the actions appear thoughtless, impulsive, and irrational.

One limitation of the integration consensus is that integration is a ubiquitous function in the nervous system, occurring for both conscious and unconscious processes. It seems that many kinds of information integration can occur unconsciously in the nervous system field. For example, in *afference binding* (Morsella and Bargh, 2011), integration occurs within sensory modalities (e.g., the binding of color to shape; Zeki and Bartels, 1999) and between sensory modalities, as in the case of the ventriloquist illusion (cf., McDonald and Ward, 2000; Watanabe and Shimojo, 2001) and in the McGurk effect (McGurk and MacDonald, 1976). (See list of unconsciously mediated intersensory illusions in Morsella, 2005, Table 1.) Unconscious integration of various kinds also occurs during motor control (the activation of muscle fibers through motor programs; James, 1890; Grossberg, 1999; Fecteau et al., 2001; Rossetti, 2001; Rosenbaum, 2002; Goodale and Milner, 2004; Johnson and Haggard, 2005; Heath et al., 2008; Liu et al., 2008), and during the control of smooth muscle (e.g., peristalsis and the pupillary reflex; Morsella et al., 2009b). Unconscious integrations also occur in the perception of the flavor of food, which involves the combining of information from multiple modalities (including haptic, gustatory, and olfactory; Shepherd, 2006), and in pain perception, in which there is, for example, interaction between sensory (lateral pain system) and affective components (medial pain system; Melzack and Casey, 1968; Nagasako et al., 2003).

Unconscious integration also occurs in *efference binding* (Haggard et al., 2002), which links perceptual processing to action/motor processing. Through this kind of stimulus-response binding, one can learn to press a button when presented with a stimulus cue in a laboratory paradigm. It has been demonstrated that, in a choice response time task, participants can select the correct motor response (one of two button presses) when confronted with subliminal stimuli (see review in Hallett, 2007). Such unconscious efference binding also takes place in the case of reflexive responses to the natural environment, as in the *pain withdrawal reflex*. [Regarding neuroanatomy, in animals such as the dog, sophisticated and intentional forms of sophisticated behavior remain when much of the cortex is removed through surgery or deactivated (e.g., chemically inactivated; Bures et al., 1974), leaving intact only the ventral forebrain, including the paleocortex (the oldest part of the forebrain), the amygdala, and the neurohumoral brain stem stimuli (Goltz, 1892; Bures et al., 1974; Panksepp, 1998). See extensive treatments in Freeman (2004) and in Merker (2007).]

In summary, the actions resulting from such unconscious bindings can seem not adaptive, as if they are not influenced by the kinds of information by which they should be influenced. Hence, these actions have been described as *un-integrated actions* (Morsella and Bargh, 2011).

As discussed in Morsella (2005), in contrast to these forms of unconscious integration, there are forms of integration that always appear to involve conscious mediation. Such is the case for *integrated actions* (Morsella and Bargh, 2011), in which *two (or more) action plans that could normally influence behavior on their own (when existing at that level of activation) are simultaneously co-activated and trying to influence the same skeletal muscle effector* (Morsella and Bargh, 2011)*.* Thus, integrated action occurs when one suppresses the urge to scratch an itch, holds one’s breath, refrains from dropping a hot dish, suppresses a pre-potent response in a laboratory paradigm, or makes oneself breathe faster (Morsella, 2005; Morsella et al., 2009a). Integrated action involves the activation of more neural processes than does un-integrated action (DeSoto et al., 2001; Ortinski and Meador, 2004). Based on these observations, it has been proposed that consciousness is required, not for just any form of integration, but for integrations involving the skeletal muscle effector system. Specifically, it has been proposed that it is required for integrating two conflicting streams of efference binding (see quantitative review of evidence in Morsella et al., 2011). Such *efference–efference binding* results in integrated actions such as holding one’s breath. Through consciousness, multiple response systems can influence behavior collectively (Morsella, 2005). Absent consciousness, only one stream can influence action control. This approach is unique in its ability to explain subjective data from (a) intersensory conflicts, which are largely unconscious, (b) smooth muscle conflicts, which, too, are largely unconscious, and (c) conflicts from action conflicts (e.g., holding one’s breath and Stroop-like interference), which tend to involve consciousness.

### CONSCIOUSNESS IS FOR VOLUNTARY ACTION

Delineating the intimate liaison between consciousness and skeletal muscle is outside the purview of the present treatise (see discussion in Morsella, 2005). For present purposes, it is important to note that this theorizing leads one to the conclusion that the integration achieved through conscious processing is intimately related – not to perceptual processing, semantic processing, smooth muscle control, or motor control – but to voluntary action. Simply put, *consciousness is for voluntary action*. In light of this, one realizes that it is no accident that, historically, skeletal muscle has been the only effector referred to as “voluntary” muscle. The appellation stems from the fact that this effector system is controlled through conscious mediation and that, without such mediation, adaptive integration fails to occur in this system, as in the case of un-integrated actions, such as reflexively dropping a hot (but expensive) dish of china or failing to hold one’s breath underwater. These are the kinds of un-integrated actions that transpire when consciousness abates. (Consistent with this approach, it has been proposed that consciousness serves to prevent premature action that does not take into account important, alternative courses of possible action, as when one avoids temptation, holds off fear and anger, or takes time to reflect on the long-term consequences of an action; Freeman, 2004.) Specifically, skeletal muscle is “voluntary” muscle because it is directed by multiple, encapsulated systems that, when in conflict, require consciousness to yield adaptive, integrated action (Morsella, 2005). For this reason, for every voluntary action emitted by the organism, the organism can self-report a conscious content that was responsible for that action (Poehlman et al., 2012), regardless of whether such an introspection is accurate or based on an illusion (Wegner, 2003). (See Freeman, 2004, for a treatment of how the notion of “circular causality” can inform theories about the function of consciousness in the nervous system.)

From this theoretical standpoint, one can hypothesize that, in olfaction, perceptual information may become conscious only once it participates in a large-scale, inter-system integration that is in the service of voluntary action, which is, stated more precisely, adaptive and integrated skeletal muscle output (see related evidence in Mainland and Sobel, 2006). By extension, one could propose (a) that, for every voluntary action based on olfactory contents, the organism can self-report about a conscious olfactory content, and (b) that, if an olfactory content is unconscious, then neither voluntary action nor integrated action can result intentionally from that content.

### THE “LOWEST HANGING FRUIT” IN THE STUDY OF THE NEURAL CORRELATES OF OLFACTORY CONSCIOUSNESS

We now conclude by reviewing what, in our review, appear to be the “lowest hanging fruit” regarding the isolation of the neural correlates of olfactory consciousness. First, regarding neuroanatomy, by synthesizing the data from various sources (including lesion studies, animal experiments, and phenomena such as blind smell and sensory habituation), investigators can determine whether peripheral structures (e.g., the olfactory epithelium and olfactory bulb) and thalamic structures (e.g., MDNT) are necessary for there to be a conscious olfactory experience of any kind, including an olfactory hallucination triggered by direct brain stimulation (e.g., in the OFC). At this stage of understanding, it seems that making such a determination would be more difficult in the case of the piriform cortex. It is important to reiterate that, if conscious olfactory experience can arise at the level of the piriform cortex, then this would be the only case in which a sensory system engenders conscious perception with little or no involvement of neocortical or thalamocortical circuits.

Second, investigators can compare the brain networks associated with (A) subliminal perception, which includes no conscious contents, (B) conscious detection of an odorant, which includes conscious contents and is indexed by self-report on the part of the subject, and (C) habituation, which contains no conscious contents. As discussed above, it remains challenging to identify the regions whose activations correspond to the phenomenal state of conscious detection versus the phenomenological nothingness of habituation. When making these contrasts, one should not be identifying the changes in neural activity associated with modulations of incentive/emotional states (e.g., hunger versus satiation). This is because, even though these states are part of the olfactory experience as a whole, they are more than just the subjective dimension of simple conscious olfactory experience. Simple conscious detection can occur (in some form) independent of these state variables. Third, if olfactory contents become conscious only when they become part of a large-scale integrative system, then what are similar kinds of integrations that can transpire *without* consciousness? Such a comparison may reveal that which is special about this form of integration. Again, it has been proposed (Morsella, 2005) that these conscious integrations differ from other forms of integration in terms of their relationship to the voluntary action system. Fourth, if olfactory consciousness cannot arise as a “microconsciousness” (Zeki and Bartels, 1999), but only when taking part in a larger-scale network, then researchers can attempt to isolate the brain rhythms associated with participation in such a network and contrast these rhythms with those occurring locally (rhythms which might not be constitutive of the conscious field).

It is our hope that, in the spirit of this special topic on *Olfactory Consciousness across Disciplines*, investigators will continue to investigate the olfactory system, the most ancient of sensory modalities, to answer these and other questions about the nature of consciousness, the most enigmatic phenomenon in nature.

## Conflict of Interest Statement

The authors declare that the research was conducted in the absence of any commercial or financial relationships that could be construed as a potential conflict of interest.
